# Inhibitory Effect of 1,5-Dimethyl Citrate from Sea Buckthorn (*Hippophae rhamnoides*) on Lipopolysaccharide-Induced Inflammatory Response in RAW 264.7 Mouse Macrophages

**DOI:** 10.3390/foods9030269

**Published:** 2020-03-02

**Authors:** Su Cheol Baek, Dahae Lee, Mun Seok Jo, Kwang Ho Lee, Yong Hoon Lee, Ki Sung Kang, Noriko Yamabe, Ki Hyun Kim

**Affiliations:** 1 School of Pharmacy, Sungkyunkwan University, Suwon 16419, Korea; schii513@daum.net (S.C.B.); anstjr920827@gmail.com (M.S.J.); sholaly@naver.com (K.H.L.); yhl2090@naver.com (Y.H.L.); 2 College of Korean Medicine, Gachon University, Seongnam 13120, Korea; pjsldh@naver.com (D.L.); kkang@gachon.ac.kr (K.S.K.)

**Keywords:** *Hippophae rhamnoides*, sea buckthorn, citric acid derivative, NO production, RAW 264.7 cells

## Abstract

*Hippophae rhamnoides* L. (Elaeagnaceae; commonly known as “sea buckthorn” and “vitamin tree”), is a spiny deciduous shrub whose fruit is used in foods and traditional medicines. The *H. rhamnoides* fruit (berry) is rich in vitamin C, with a level exceeding that found in lemons and oranges. *H. rhamnoides* berries are usually washed and pressed to create pomace and juice. Today, the powder of the aqueous extract of *H. rhamnoides* berries are sold as a functional food in many countries. As part of our ongoing effort to identify bioactive constituents from natural resources, we aimed to isolate and identify those from the fruits of *H. rhamnoides*. Phytochemical analysis of the extract of *H. rhamnoides* fruits led to the isolation and identification of six compounds, namely, a citric acid derivative (**1**), a phenolic (**2**), flavonoids (**3** and **4**), and megastigmane compounds (**5** and **6**). Treatment with compounds **1**–**6** did not have any impact on the cell viability of RAW 264.7 mouse macrophages. However, pretreatment with these compounds suppressed lipopolysaccharide (LPS)-induced NO production in RAW 264.7 mouse macrophages in a concentration-dependent manner. Among the isolated compounds, compound **1** was identified as the most active, with an IC_50_ of 39.76 ± 0.16 μM. This value was comparable to that of the *N*^G^-methyl-L-arginine acetate salt, a nitric oxide synthase inhibitor with an IC_50_ of 28.48 ± 0.05 μM. Western blot analysis demonstrated that compound **1** inhibited the LPS-induced expression of IKKα/β (IκB kinase alpha/beta), I-κBα (inhibitor of kappa B alpha), nuclear factor kappa-B (NF-κB) p65, iNOS (inducible nitric oxide synthase), and COX-2 (cyclooxygenase-2) in RAW 264.7 cells. Furthermore, LPS-stimulated cytokine production was detected using a sandwich enzyme-linked immunosorbent assay. Compound **1** decreased interleukin 6 (IL-6) and tumor necrosis factor alpha (TNF-α) production in LPS-stimulated RAW 264.7 cells. In summary, the mechanism of action of **1** included the suppression of LPS-induced NO production in RAW 264.7 cells by inhibiting IKKα/β, I-κBα, NF-κB p65, iNOS, and COX-2, and the activities of IL-6 and TNF-α.

## 1. Introduction

*Hippophae rhamnoides* L. (commonly known as ‘Sea buckthorn’ and ‘Vitamin tree’) is a spiny deciduous shrub belonging to the Elaeagnaceae family. The fruit of *H. rhamnoides* is rich in vitamin C and its levels exceed those found in lemons and oranges [1]. The berries of *H. rhamnoides* are usually washed and pressed to create pomace and juice. In many countries, the powder from the aqueous extract of *H. rhamnoides* berries are consumed as a functional food. The berry of *H. rhamnoides* is used as food and medicine in Asia, specifically in Tibetan and Mongolian medicines, owing to its antibechic, antiviral, and antioxidant activities [2,3]. Moreover, the berries are used to make fruit sauce, fruit powder, and fruit wine [4]. Previous biological studies of the *H. rhamnoides* extracts revealed their anti-platelet effect through the inhibition of thrombin-activated platelets to collagen or fibrinogen [5]. The phenolic and nonpolar lipid fractions of the *H. rhamnoides* extracts attenuate the essential pathogenic properties of *Staphylococcus aureus* and *Candida albicans*, and the mechanism of the effect was found to be due to a decrease in adhesion and biofilm formation on inert surfaces [6]. Previously, a study found that the pomace of *H. rhamnoides* exerts antioxidant activity [7]. Therefore, because of the health benefits of *H. rhamnoides*, its phytochemical constituents were investigated, and further demonstrated to include flavonoids, alkaloids, protocatechuic acid, and triterpenoid glycosides [8,9,10]. Among them, isorhamnetin 3,5,7,4-tetrahydroxy-3-methoxyflavon, isorhamnetin 3-*O*-*β*-D-glucopyranosil-(6-1)-*O*-*α*-1-rhamnopyranoside, and protocatechuic acid were found to have a regulatory effect on hepatic stellate cell activation and liver fibrogenesis by affecting changes in DNA expression and cell cycle arrest, and decreasing the release of cytokines [11].

As natural products are rich sources of anti-inflammatory agents [12,13], evaluating their anti-inflammatory effects using laboratory models is important for drug discovery. Inflammation is a critical immune response to different factors, such as bacteria, chemicals, and viruses, that result in inflammation-mediated diseases, including inflammatory bowel disease, cancer, atherosclerosis, diabetes, and asthma [14]. Discovering the bioactive constituents present in natural products can thus serve as an approach to prevent and treat inflammation-mediated diseases. Numerous laboratory models are available for screening and assessing the anti-inflammatory agents derived from natural products [12]. Through in vitro and in vivo laboratory models, macrophages have been identified as the principal cell type in the innate immune system [15]. Lipopolysaccharides (LPS), which are known as an endotoxin of Gram-negative bacteria, have been widely used to study the inflammatory response in macrophages. This is because activated macrophages release NO as an indicator of pro-inflammatory reactions in response to LPS [16,17]. During the above process, the nuclear factor kappa-B (NF-κB) proteins translocate to the nucleus from the cytoplasm and induce the transcription of pro-inflammatory mediators and cytokines [18]. Thus, the ability of NF-κB to modulate the expression of cyclooxygenase-2 (COX-2), inducible nitric oxide synthase (iNOS), interleukin 6 (IL-6), and tumor necrosis factor alpha (TNFα) allows us to assess the anti-inflammatory effect of natural products against inflammatory response.

As part of our ongoing research to discover the bioactive constituents in natural sources [19,20,21,22], here we elucidated the bioactive compounds in the fruits of *H. rhamnoides*. Through a phytochemical analysis of the extract from *H. rhamnoides* fruits, we isolated and identified six compounds, including a citric acid derivative (**1**), a phenolic (**2**), two flavonoids (**3** and **4**), and two megastigmane compounds (**5** and **6**), through a comparison of their nuclear magnetic resonance (NMR) spectroscopic data to their reported values and the results of LC/MS analysis. In the present study, we sought to report the anti-inflammatory effect of the constituents of *H. rhamnoides* and elucidate the underlying molecular mechanism whereby the active compound **1** modulates the NF-κB p65 signaling pathway, which affects the LPS-induced inflammatory responses in RAW 264.7 mouse macrophages.

## 2. Materials and Methods

### 2.1. General Experimental Procedures

Ultraviolet (UV) spectra were acquired on an Agilent 8453 UV-visible spectrophotometer (Agilent Technologies, Santa Clara, CA, USA). Experimental electronic circular dichroism (ECD) spectra in methanol (MeOH) were acquired in a quartz cuvette of 1 mm optical path length on a JASCO J-1500 spectropolarimeter (Tokyo, Japan). Nuclear magnetic resonance (NMR) spectra were recorded on a Bruker AVANCE III HD 850 NMR spectrometer with a 5 mm TCI CryoProbe operated at 850 MHz (^1^H) and 212.5 MHz (^13^C) (Bruker, Karlsruhe, Germany). For the ^1^H and ^13^C NMR analyses, chemical shifts are expressed in ppm (δ). Medium-pressure liquid chromatography (MPLC) was conducted using Smart Flash AKROS (Yamazen, Osaka, Japan). Semi-preparative HPLC was carried out using a Shimadzu Prominence HPLC System with SPD-20A/20AV Series Prominence HPLC UV-VIS detectors (Shimadzu, Tokyo, Japan) and a Phenomenex Luna C18 column (250 × 10 mm, 5 μm; flow rate: 2 mL/min; Phenomenex, Torrance, CA, USA). LC/MS analysis was performed on an Agilent 1200 Series HPLC system equipped with a diode array detector, a 6130 Series ESI mass spectrometer, and an analytical Kinetex C18 100 Å column (100 × 2.1 mm, 5 μm; flow rate: 0.3 mL/min; Phenomenex). Silica gel 60 (230–400 mesh; Merck, Darmstadt, Germany) and RP-C_18_ silica gel (Merck, 230–400 mesh) were used for column chromatography. Sephadex LH-20 (Pharmacia, Uppsala, Sweden) was employed as the packing material for molecular sieve column chromatography. Merck precoated silica gel F_254_ plates and RP-C_18_ F_254s_ plates were used for thin-layer chromatography (TLC). Following TLC, spots were detected under UV light or by heating when sprayed with anisaldehyde-sulfuric acid.

### 2.2. Plant Material

The powder of *H. rhamnoides* fruits was purchased in October of 2018 from Korea Beauty and Healthcare Co., Ltd. Thereafter, the material was identified by one of the authors (K.H.K). A voucher specimen of the material (VT-2018) was deposited in the herbarium at the School of Pharmacy, Sungkyunkwan University, Suwon, Republic of Korea.

### 2.3. Extraction and Isolation

The aqueous extract powder (270 g) of *H. rhamnoides* fruits was suspended in distilled water (700 mL) prior to solvent partitioning with *n*-hexane, dichloromethane (CH_2_Cl_2_), ethyl acetate (EtOAc), and *n*-butanol (BuOH) to obtain four partitioned layers: hexane-soluble (2.7 g), methylene chloride (MC)-soluble (3.4 g), ethyl acetate (EA)-soluble (7.8 g), and BuOH-soluble (25.2 g) layers. The MC-soluble layer (3.4 g) was subjected to silica gel column chromatography (100 g, eluted with MC/MeOH (100:1 ⟶ 1:1); gradient solvent system, washed with 100% MeOH) to yield seven fractions (C1-C7). Fraction C4 (55.7 mg) was separated via MPLC on a Yamazen UNIVERSAL Premium silica column with MC/MeOH (100:0 ⟶ 1:1) to yield four subfractions (C41–C44). Subfraction C41 (46.4 mg) was separated on a Sep-pak C18 cartridge using a solvent system consisting of 90% MeOH to yield three subfractions (C411–C413). Subfraction C411 (17.4 mg) was subsequently purified by semi-preparative HPLC (35% MeOH) on a Phenomenex Luna C18 column to yield compound **5** (*t*_R_ 36.5 min, 0.5 mg). Fraction C5 (74.5 mg) was purified by semi-preparative HPLC (39% MeOH) on a Phenomenex Luna C18 column to yield compound **6** (*t*_R_ 23.0 min, 1.5 mg). The EA-soluble layer (7.8 g) was chromatographed by silica gel column chromatography (200 g, eluted with MC/MeOH (20:1 ⟶ 1:1); gradient solvent system, washed with 100% MeOH) to yield seven fractions (E1-E7). Fraction E3 (751.1 mg) was separated on a Sephadex LH-20 column using a solvent system of 100% MeOH to yield five subfractions (E31–E35). Subfraction E35 (15.7 mg) was purified by semi-preparative HPLC (19% acetonitrile) on a Phenomenex Luna C18 column to yield compound **2** (*t*_R_ 34.8 min, 1.0 mg). Fraction E4 (1.37 g) was separated on a Sephadex LH-20 column using a solvent system of 100% MeOH to yield three subfractions (E41-E43). Subfraction E42 (1.24 g) was subjected to silica gel column chromatography (100 g, eluted with MC/MeOH (20:1 ⟶ 1:1); gradient solvent system, washed with 80% MeOH) to yield six subfractions (E421–E426). Subfraction E422 (391.3 mg) was separated by reversed-phase preparative HPLC on an Agilent Eclipse C18 column with a gradient solvent system of MeOH/H_2_O (10%–70%–100%) to yield five subfractions (E4221–E4225). Subfraction E4223 was purified by semi-preparative HPLC (20% MeOH) on a Phenomenex Luna C18 column to yield subfraction E42236. Subfraction E42236 (23.0 mg) was re-purified by semi-preparative HPLC (10% MeOH) on a Phenomenex Luna C18 column to yield compound **1** (*t*_R_ 37.5 min, 6.0 mg). Subfraction E43 (55.3 mg) was separated by semi-preparative HPLC (15% MeOH) to yield six subfractions (E431–E436). Subfraction E436 was purified by semi-preparative HPLC (38% MeOH) on a Phenomenex Luna C18 column to yield compounds **3** (*t*_R_ 56.6 min, 0.6 mg) and **4** (*t*_R_ 58.5 min, 0.9 mg).

### 2.4. RAW 264.7 Cell Culture

The mouse macrophage cell line, RAW 264.7 (American Type Culture Collection, Rockville, MD, USA), was cultured in Dulbecco’s modified Eagle’s medium (Cellgro, VA, USA) containing 10% fetal bovine serum (FBS) and 1% penicillin/streptomycin (Invitrogen Co., NY, USA) at 37 °C in an atmosphere of 5% CO_2_.

### 2.5. Measurement of RAW 264.7 Cell Viability

RAW 264.7 cell viability was determined using an Ez-Cytox cell viability detection kit (Daeil Lab Service Co., Seoul, Korea). Cells were plated in 96-well plates at a density of 3 × 10^4^ cells/well and incubated for 24 h at 37 °C. Cells were exposed to compounds at concentrations of 5, 10, 25, 50, and 100 μM for 1 h at 37 °C. Thereafter, they were incubated with vehicle (medium containing 0.5% DMSO) for 24 h at 37 °C. After that, they were incubated with the Ez-Cytox solution for 40 min at 37 °C. Absorbance was determined at 450 nm using a PowerWave XS microplate reader (Bio-Tek Instruments, Winooski, VT, USA).

### 2.6. Measurement of NO Production in RAW 264.7 Cells

RAW 264.7 cells were plated in 96-well plates at a density of 3 × 10^4^ cells/well for a 24 h incubation at 37 °C. Cells were either exposed to the compounds or *N*^G^-methyl-L-arginine acetate salt (L-NMMA) at concentrations of 5, 10, 25, 50, and 100 μM for 1 h at 37 °C. Thereafter, they were incubated with 1 μg/mL LPS for 24 h at 37 °C. The supernatant was mixed with an equal volume of Griess reagent containing 0.2% naphthylethylenediamine dihydrochloride (Sigma-Aldrich, St. Louis, MO, USA) and 2% sulfanilamide (Sigma-Aldrich) in 5% phosphoric acid (Sigma-Aldrich) and incubated for 40 min at 37 °C. The absorbance was then measured at 540 nm using a PowerWave XS microplate reader.

### 2.7. Western Blot Analysis

RAW 264.7 cells were plated in 6-well plates at a density of 2 × 10^5^ cells/well and incubated for 24 h at 37 °C. Cells were exposed to 50 and 100 μM of compound **1** for 1 h at 37 °C prior to incubation with 1 μg/mL LPS for 24 h at 37 °C. Whole-cell extracts were then prepared using radio immunoprecipitation assay buffer (RIPA buffer; Cell Signaling Technology, Inc., Beverly, MA, USA) containing 1× ethylenediaminetetraacetic acid (EDTA)-free protease inhibitor cocktail and 1 mM phenylmethylsulfonyl fluoride (PMSF), according to the manufacturer’s instructions. Protein concentration of the whole-cell extracts was determined with a bicinchoninic acid (BCA) protein assay kit. Proteins (20 μg/lane) were subjected to 10% sodium dodecyl sulfate polyacrylamide gel electrophoresis (SDS–PAGE) for 90 min at 110 V, transferred to a polyvinylidene fluoride (PVDF) membrane, and analyzed with epitope-specific primary antibodies to phospho-IκB kinase (IKK)α/β, IKKα (IκB kinase alpha), IKKβ (IκB kinase beta), I-κBα (inhibitor of kappa B alpha), phospho-I-κBα, phospho-NF-κB p65, NF-κB p65, and iNOS, followed by cyclooxygenase-2 (COX-2), glyceraldehyde 3-phosphate dehydrogenase (GAPDH), and horseradish peroxidase (HRP)-conjugated anti-rabbit antibodies (Cell Signaling, MA, USA). The bound antibodies were then visualized with ECL Advance Western Blotting Detection Reagents (GE Healthcare, Cambridge, United Kingdom) and a FUSION Solo Chemiluminescence System (PEQLAB Biotechnologie GmbH, Erlangen, Germany). The optical densities of the immunoreactive bands were obtained using ImageJ software (Version 1.51J; National Institutes of Health, Bethesda, MD, USA) and normalized with those of the control. Data represented by fold-increases were compared to the control. The GAPDH was used for loading control.

### 2.8. Determination of IL-6 and TNF-α Production

RAW 264.7 cells were plated in 24-well plates at a density of 4 × 10^5^ cells/well and incubated for 24 h at 37 °C. Cells were then exposed to compound **1** at concentrations of 50 and 100 μM for 1 h at 37 °C prior to incubation with 1 μg/mL LPS for 24 h at 37 °C. To determine IL-6 and tumor necrosis factor alpha (TNF-α) production, the supernatant was obtained and assayed to quantify the levels of these cytokines using an IL-6 sandwich enzyme-linked immunosorbent assay (ELISA) kit (BD Biosciences, CA, USA) and a TNF-α ELISA kit (eBiosciences, CA, USA), according to the manufacturer’s instructions.

### 2.9. Statistical Analysis

At least three independent determinations were carried out for each experiment. All data are presented as average value and standard deviation (SD). Statistical significance was determined using one-way analysis of variance (ANOVA), and multiple comparisons were carried out with a Bonferroni correction. A *p*-value less than 0.05 indicated statistical significance. All statistical analyses were performed using SPSS Statistics version 19.0 (SPSS Inc., Chicago, IL, USA).

## 3. Results and Discussion

### 3.1. Isolation and Identification of Compounds

**Figure 1:**
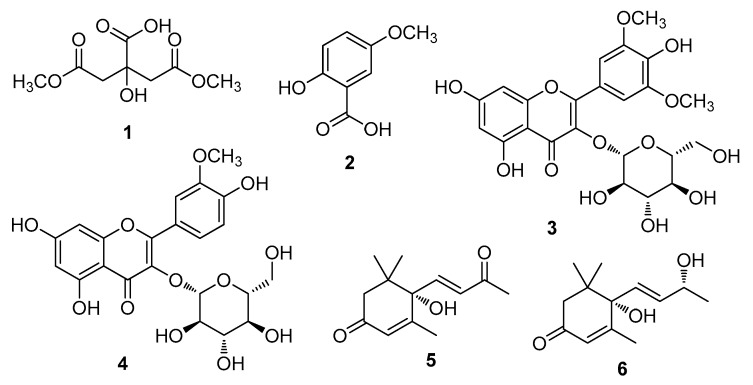
Chemical structures of compounds **1–6**. 1,5-Dimethyl citrate (**1**), 5-methoxysalicylic acid (**2**), syringetin-3-*O*-glucoside (**3**), isorhmanetin-3-*O*-glucoside (**4**), (+)-dehydrovomifoliol (**5**), and (+)-vomifoliol (**6**).

The aqueous extract powder of *H. rhamnoides* fruits was first suspended in distilled water, and then subjected to solvent-partitioning with *n*-hexane, CH_2_Cl_2_, ethyl acetate, and *n*-BuOH to obtain each solvent fraction. Phytochemical analysis of the CH_2_Cl_2_-soluble and ethyl acetate-soluble fractions was performed using repeated column chromatography, HPLC, and LC/MS. Through chemical analysis, six compounds (**1**–**6**), including a citric acid derivative (**1**), a phenolic (**2**), two flavonoids (**3** and **4**), and two megastigmane compounds (**5** and **6**) (Figure 1) were isolated; compounds **1**-**4** were isolated from the ethyl acetate-soluble fraction whereas compounds **5**-**6** were isolated from the CH_2_Cl_2_-soluble fraction. By comparing the NMR spectroscopic data of the compounds to their reported values and the LC/MS results, we identified that the compounds were 1,5-dimethyl citrate (**1**) [23], 5-methoxysalicylic acid (**2**) [24], syringetin-3-*O*-glucoside (**3**) [25], isorhamnetin-3-*O*-glucoside (**4**) [9], (+)-dehydrovomifoliol (**5**) [26], and (+)-vomifoliol (**6**) [27]. In particular, the absolute configurations for compounds **5** and **6** were established by their positive specific rotation values and comparison of their NMR spectroscopic data with those reported earlier and electronic circular dichroism (ECD) data (Figure S1) [26,27].

### 3.2. Effects of Compounds **1**–**6** on Cell Viability

**Figure 2:**
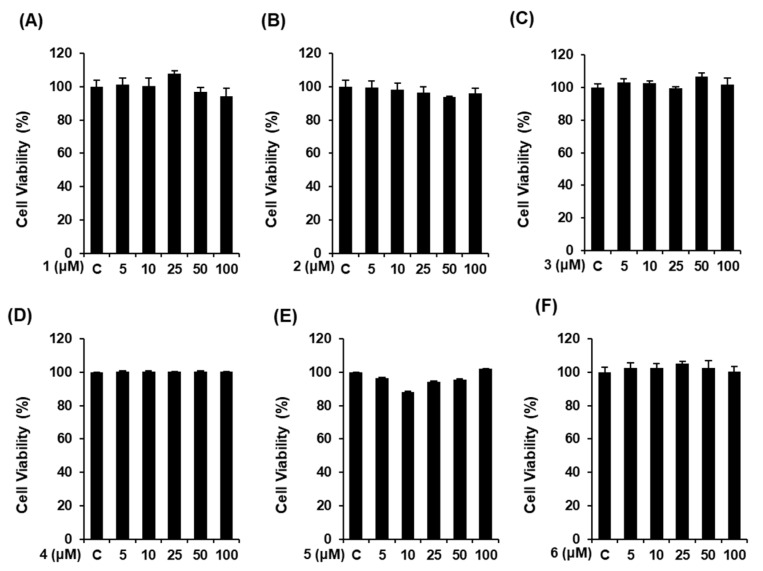
Effect of compounds **1**–**6** on the viability of RAW 264.7 mouse macrophages. (**A**–**F**) The viability of RAW 264.7 cells incubated with compounds **1–6** for 24 h was measured using an Ez-Cytox cell viability assay (mean ± SD, * *p* < 0.05 compared to the lipopolysaccharide (LPS)-treated group). C: control group treated with 0.5% DMSO. 1,5-dimethyl citrate (**1**), 5-methoxysalicylic acid (**2**), syringetin-3-*O*-glucoside (**3**), isorhmanetin-3-*O*-glucoside (**4**), (+)-dehydrovomifoliol (**5**), and (+)-vomifoliol (**6**).

Prior to evaluating the effect of the compounds on NO production, we determined their cytotoxicity in RAW 264.7 mouse macrophages using an Ez-Cytox cell viability assay. As a result, the compounds were considered to be cytotoxic when the cell viability in the compound-treated group was less than 80% of that in the untreated group. At all tested concentrations, all concentrations of compounds **1**–**6** had no cytotoxicity on RAW 264.7 mouse macrophages (Figure 2). Therefore, all concentrations were employed for the subsequent experiments.

### 3.3. Effects of Compounds **1**–**6** on NO Production

**Figure 3:**
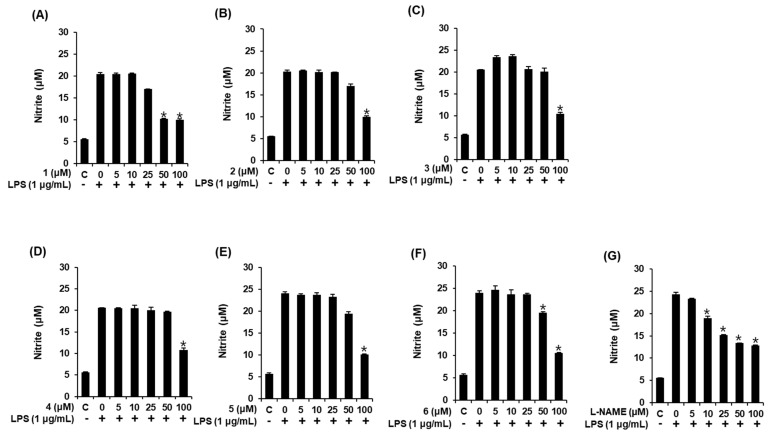
Effect of compounds **1**–**6** on the LPS-stimulated NO production in RAW 264.7 mouse macrophages. The effect of (**A**–**F**) the compounds and (**G**) *N*^G^-methyl-L-arginine acetate salt (L-NMMA) in LPS-treated RAW 264.7 macrophages was detected using the Griess reagent (mean ± SD, * *p* < 0.05 compared to the LPS-treated group). C: control group treated with 0.5% DMSO. 1,5-Dimethyl citrate (**1**), 5-methoxysalicylic acid (**2**), syringetin-3-*O*-glucoside (**3**), isorhmanetin-3-*O*-glucoside (**4**), (+)-dehydrovomifoliol (**5**), and (+)-vomifoliol (**6**).

The production of NO in LPS-induced RAW 264.7 macrophages has been used as an indicator to evaluate the anti-inflammatory effects of natural products. In the present study, we evaluated the effect of compounds **1–6** on LPS-induced NO production in RAW 264.7 cells. In this assay, L-NMMA, a nitric oxide synthase inhibitor, was used as the positive control. As shown in Figure 3, exposure to 1 μg/mL LPS induced a significant increase in NO production; however, this was suppressed by pretreatment with compounds **1–6**, which had 50% inhibitory concentration (IC_50_) values of 39.76 ± 0.16 μM, 79.39 ± 0.29 μM, 86.33 ± 0.54 μM, 87.01 ± 0.30 μM, 74.71 ± 0.24 μM, and 76.12 ± 0.14 μM, respectively. Although the IC_50_ values of most compounds were slightly weaker than that of L-NMMA (IC_50_, 28.48 ± 0.05 μM), compound **1** displayed a similar IC_50_ value, which indicates that it might inhibit the increase of LPS-induced NO production in RAW 264.7 macrophages. This finding suggests that the inflammatory responses induced by extracellular stimuli might be suppressed via treatment with compound **1**. Thus, we proceeded to investigate the potential benefit of compound **1** as an NO production inhibitor by carrying out a mechanism study.

### 3.4. Compound **1** Downregulated IKKα/β, I-κBα, and NF-κB p65 in LPS-Stimulated RAW 264.7 Mouse Macrophages

**Figure 4:**
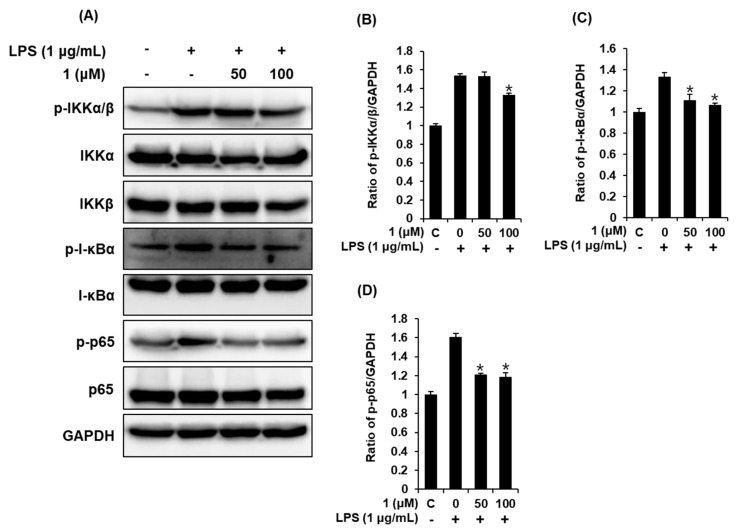
Effects of compound **1** on the LPS-induced expression of the IKKα/β (IκB kinase alpha/beta), I-κBα (inhibitor of kappa B alpha), and NF-κB p65 proteins in RAW 264.7 mouse macrophages. (**A**) Representative Western blots of IKKα/β, I-κBα, NF-κB p65, and glyceraldehyde 3-phosphate dehydrogenase (GAPDH) protein expression. Quantitative graph of (**B**) p-IKKα/β, (**C**) p-I-κBα, and (**D**) p-p65 (mean ± SD, * *p* < 0.05 compared to the LPS-treated group). C: control group treated with 0.5% DMSO. 1,5-dimethyl citrate (**1**).

When macrophages are stimulated with LPS, IKKα/β is phosphorylated, thereby leading to the subsequent phosphorylation and degradation of IκBα, which enables the nuclear translocation of NF-κB [28]. NF-κB p65 is a core mediator of the innate immune response that leads to the transcription of genes encoding pro-inflammatory mediators, such as iNOS and COX-2, and pro-inflammatory cytokines, such as TNF-α and IL-6 [29,30]. Therefore, to confirm whether pre-treatment with compound **1** could suppress LPS-stimulated NO production through the NF-κB p65 signaling pathway, we determined the effect of compound **1** on the expression of IKKα/β, I-κBα, and NF-κB p65 in LPS-stimulated RAW 264.7 mouse macrophages. As shown in Figure 4, exposure to 1 μg/mL LPS significantly upregulated IKKα/β, I-κBα, and NF-κB p65; however, pretreatment with 50 and 100 μM of compound **1** reversed these changes induced by LPS. Such findings indicate that compound **1** inhibits NO production by inhibiting the phosphorylation of IKKα/β, I-κBα, and NF-κB p65 in RAW 264.7 macrophages. On the basis of these results, it appears that the action site of compound **1** is upstream of the IKK site, thus modulating inflammatory mediator expression.

### 3.5. Compound **1** downregulated iNOS and COX-2 expression in LPS-stimulated RAW 264.7 mouse macrophages

**Figure 5:**
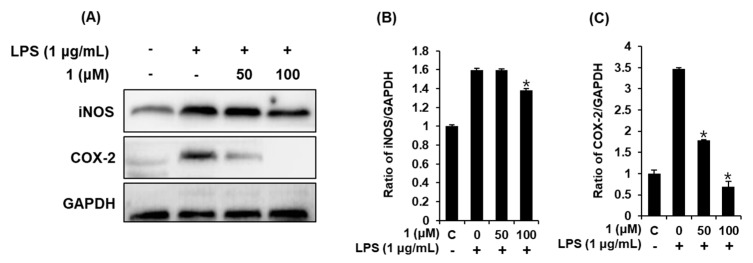
Effects of compound **1** on the LPS-induced expression of iNOS and COX-2 in RAW 264.7 mouse macrophages. (**A**) Representative Western blots of iNOS, COX-2, and GAPDH protein expression. Quantitative graph of (**B**) iNOS and (**C**) COX-2 (mean ± SD, * *p* < 0.05 compared to the LPS-treated group). C: control group treated with 0.5% DMSO. 1,5-dimethyl citrate (**1**).

The induction of iNOS and COX-2 during inflammation directly contributes to NO synthesis [31]. Therefore, we proceeded to verify whether compound **1** (1,5-dimethyl citrate) affects LPS-stimulated iNOS and COX-2. As shown in Figure 5, exposure to 1 μg/mL LPS significantly upregulated iNOS and COX-2; however, pretreatment with 50 and 100 μM of compound **1** could reverse the changes induced by LPS. Compound **1** could therefore inhibit the increase in iNOS and COX-2 induced via the NF-κB signaling pathway. On the basis of these results, the suppression in NO production of compound **1** might be associated with downregulating the expression of iNOS and COX-2 by LPS stimulation in RAW 264.7 macrophages.

### 3.6. Compound **1** downregulated IL-6 and TNF-α production in LPS-stimulated RAW 264.7 mouse macrophages

**Figure 6:**
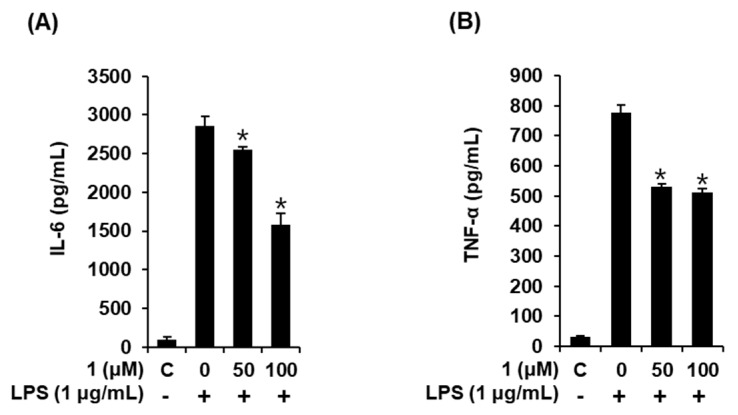
Effects of compound **1** on the LPS-induced cytokine release in RAW 264.7 mouse macrophages. (**A**) Effect of compound **1** on LPS-induced IL-6 production. (**B**) Effects of compound **1** on LPS-induced TNF-α production (mean ± SD, * *p* < 0.05 compared to the LPS-treated group). C: control group treated with 0.5% DMSO. 1,5-dimethyl citrate (**1**).

**Figure 7:**
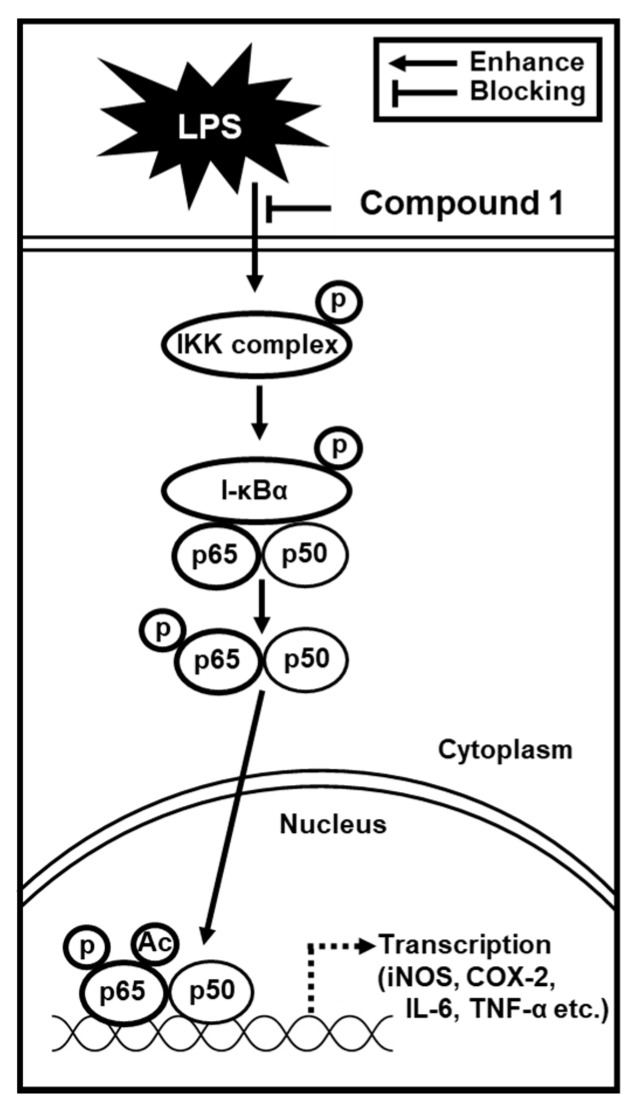
Schematic model showing the inhibitory effects of compound **1** on lipopolysaccharide-induced inflammatory response in RAW 264.7 mouse macrophages through the inhibition of IKKα/β, I-κBα, NF-κB p65, inducible nitric oxide synthase (iNOS), and cyclooxygenase-2 (COX-2), and the activities of IL-6 and TNF-α. 1,5-dimethyl citrate (**1**).

Elevated expression levels of iNOS and COX-2 followed by an increase in the production of NO have been found to be evoked by TNF-α and IL-6 in RAW 264.7 macrophages [32]. Lastly, we proceeded to examine whether compound **1** affected the production of pro-inflammatory cytokines, including IL-6 and TNF-α, in LPS-stimulated RAW 264.7 mouse macrophages. As shown in Figure 6, treating RAW 264.7 macrophages with LPS markedly increased IL-6 and TNF-α production, which were ameliorated by 50 and 100 μM of compound **1** in a concentration-dependent manner. These results suggest that compound **1** inhibits IL-6 and TNF-α production, ultimately leading to a reduction in inflammatory response. Altogether, we found that the inhibition of NO production in LPS-activated RAW 264.7 macrophages by compound **1** was mediated by the inhibition of IKKα/β, I-κBα, NF-κB p65, iNOS, and COX-2, as well as the activities of IL-6 and TNF-α (Figure 7).

In previous studies, flavonoids as main components of sea buckthorn (*H. rhamnoides*) including isorhamnetin, quercetin, and kaempferol, inhibited LPS-induced NO production in RAW 264.7 cells through the inhibition of expressions of iNOS, COX-2, proteins in the mitogen-activated protein kinase (MAPK) pathway (c-Jun N-terminal kinase and p38), I-κBα, NF-κB p65, and production of pro-inflammatory cytokines (IL-6, IL-1β, and TNF-α) [33]. The mechanism of action of flavonoids from sea buckthorn was similar to that of 1,5-dimethyl citrate (**1**) that we identified in this study. However, it has not been known exactly which flavonoid of sea buckthorn exhibits the anti-inflammatory effect. To the best of our knowledge, this study is the first to evaluate the anti-inflammatory activity of citric acid derivative isolated from sea buckthorn on LPS-induced inflammatory response in RAW 264.7 mouse macrophages. The active compound, 1,5-dimethyl citrate (**1**), was a simple derivative of citric acid present in many plants and fruits [34,35,36,37]. An anti-inflammatory effect of citric acid derivatives has been reported in previous studies [34,35,36,37]. In LPS-treated mice, increased TNF-α in mice brain tissue and iNOS expression in the cytoplasm of hepatocytes were decreased after treatment of citric acid [34]. In a recent study, wheat germ extract with citric acid was reported to inhibit secretion of the pro-inflammatory cytokines, TNF-α, IL-6, and IL-12 and the synthesis of COX-2, as well as phosphorylation of NF-κB p65 and p38 kinase in LPS-activated macrophages [35]. In addition, calcium citrate showed antioxidant enzyme activities, and inhibited NO production by suppression of the expression of pro-inflammatory mediators (NF-κB, iNOS, and COX-2) and cytokines (TNF-α, IL-1β, and IL-6) in LPS-stimulated RAW 264.7 macrophages [36]. Another recent study reported that citric acid displayed anti-inflammatory function via the toll-like receptor (TLR)-mediated activation of NF-κB and interferon regulatory factor-3 signaling pathways, and that citric acid contributed to the maintenance of tight junction proteins via the TLR-mediated p38 and c-Jun N-terminal kinase pathways [37]. Interestingly, a new oligosaccharide citric acid derivative isolated from *Vigna angularis* (ohwi *et* ohashi. var. Dainagon) seeds displayed promising anti-influenza A virus activity [38]. Additionally, a recent paper reported that citric acid showed protective effect against hepatic ischemia reperfusion injury in Sprague-Dawley rats, which suggests significant therapeutic potential of citric acid in ischemic liver injury [39]. Although future in vivo studies should support the anti-inflammatory effects of 1,5-dimethyl citrate (**1**), identified as an active compound in this study, our findings suggest a potential application of 1,5-dimethyl citrate (**1**) for the treatment of inflammatory diseases.

## 4. Conclusions

Herein, we provided experimental evidence of the potential role of 1,5-dimethyl citrate from *H. rhamnoides* fruits in the management and treatment of inflammatory diseases. By performing a phytochemical analysis of the extracts of *H. rhamnoides* fruits using repeated column chromatography, HPLC, and LC/MS, we isolated and identified six compounds (**1–6**), namely, a citric acid derivative (**1**), a phenolic (**2**), two flavonoids (**3** and **4**), and two megastigmane compounds (**5** and **6**). Among them, 1,5-dimethyl citrate (**1**) was demonstrated as effectively preventing LPS-induced NO production and markedly inhibited the expression of IKKα/β, I-κBα, NF-κB p65, iNOS, and COX-2, and the activities of IL-6 and TNF-α. On the basis of our findings, we have provided experimental evidence that the 1,5-dimethyl citrate (**1**) obtained from *H. rhamnoides* fruits could function as an effective agent for the treatment of inflammatory diseases.

## Supplementary Materials

The following are available online at https://www.mdpi.com/2304-8158/9/3/269/s1: Figure S1: ECD data of compounds 5 (A) and 6 (B), Figure S2: LC/MS-based analysis (detection wavelength was set as 210 nm) of the ethyl acetate-soluble fraction as well as the chemical structures of compounds 1–4, Figure S3: LC/MS-based analysis (detection wavelength was set as 210 nm) of the CH2Cl2-soluble fraction as well as the chemical structures of compounds 5–6, Figure S4: LC/MS analysis for purity of compounds 1–6.
