# Supplementary material for: Inhibitory Effect of 1,5-Dimethyl Citrate from Sea Buckthorn (Hippophae rhamnoides) on Lipopolysaccharide-Induced Inflammatory Response in RAW 264.7 Mouse Macrophages
Source: Foods. 2020 Mar 2;9(3):269. doi: 10.3390/foods9030269 (PMC7143571; doi:10.3390/foods9030269)
Supplement: Supplementary file 1 [file foods-09-00269-s001.pdf]

Supplementary Materials

Figure S1. ECD data of compounds **5** (A) and **6** (B).

(A)

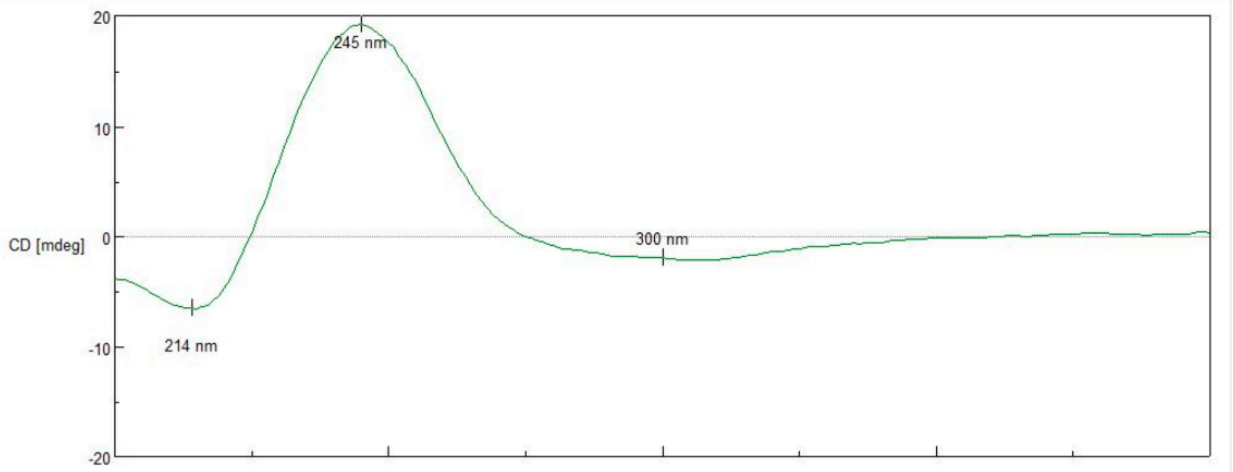

(B)

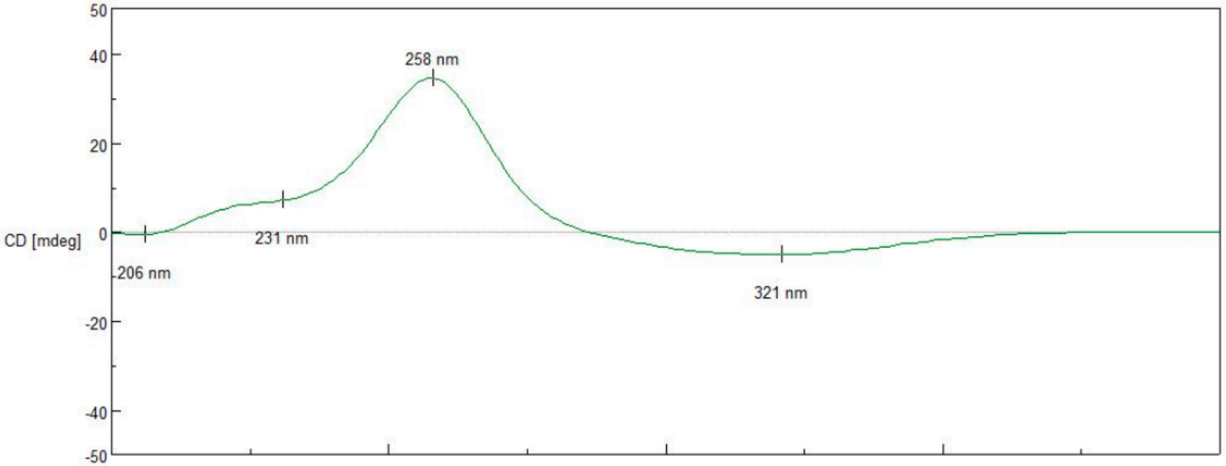

Figure S2. LC/MS-based analysis (detection wavelength was set as 210 nm) of the ethyl acetate-soluble fraction as well as the chemical structures of compounds **1–4**.

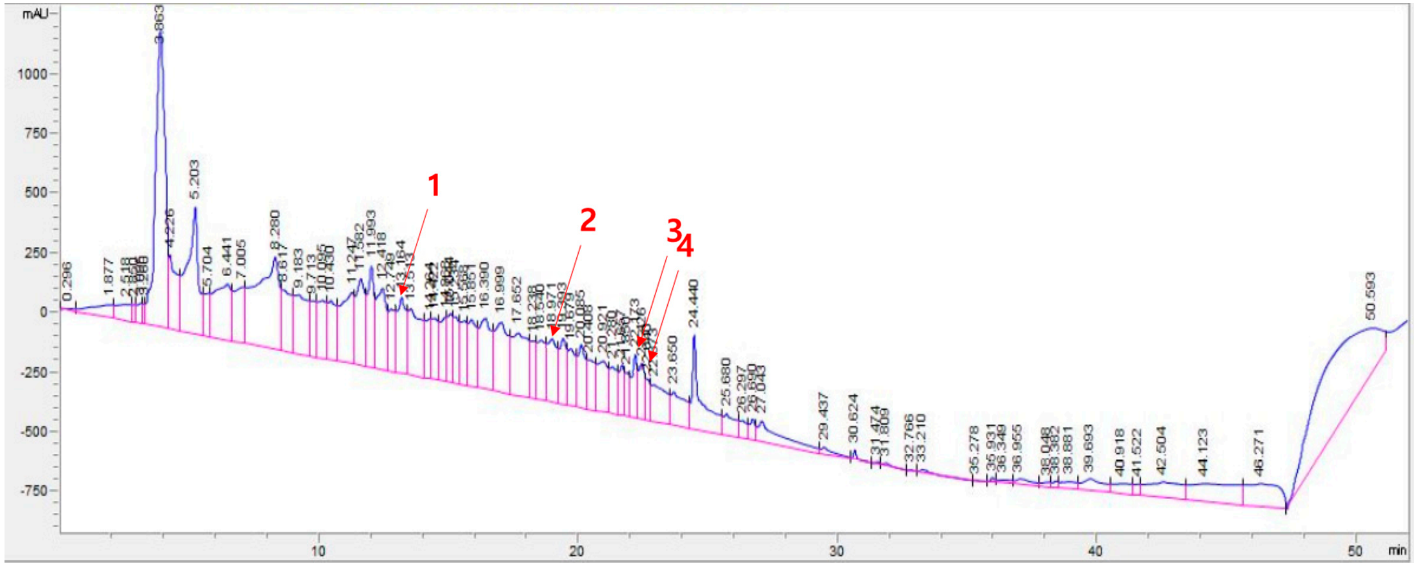

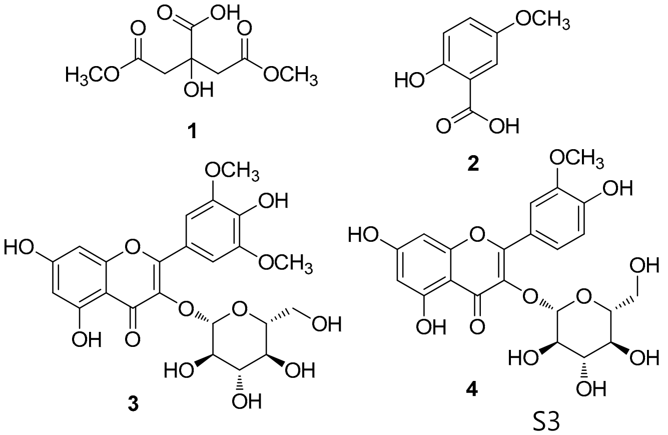

Analysis condition
- Agilent Technologies 1200s
- Column: Agilent Eclipse Plus C18 3.5 μm 100 x 4.6 mm
- Flow: 0.3 mL/min
- Detector: PDA detector (210 nm)
- Mobile phase: Water-MeOH gradient

Time	MeOH
0.0	10
30	100
40	100
50	10

Figure S3. LC/MS-based analysis (detection wavelength was set as 210 nm) of the CH_2Cl_2-soluble fraction as well as the chemical structures of compounds **5–6**.

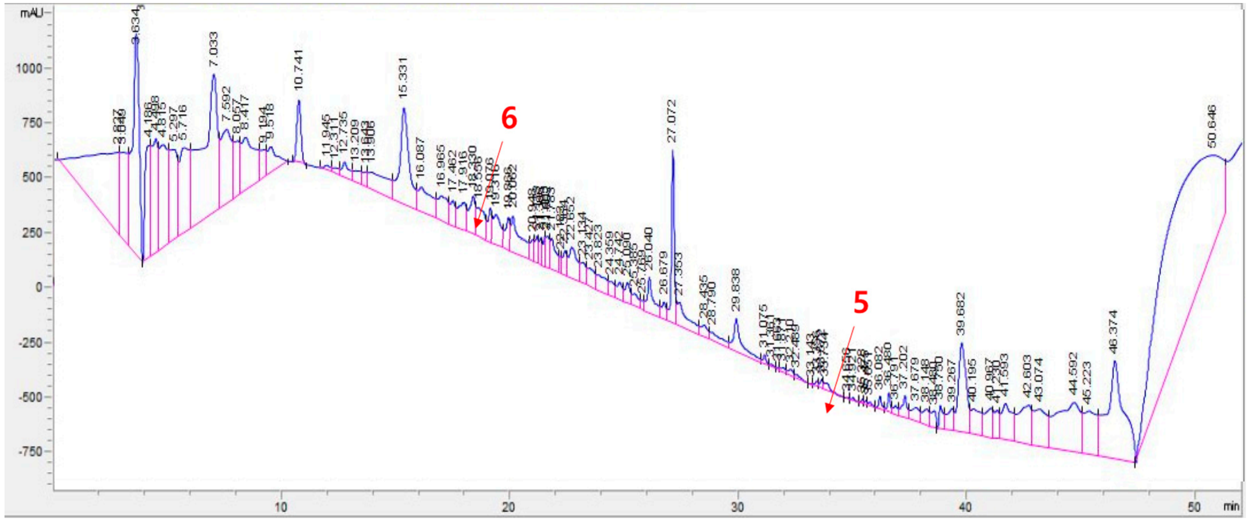

Analysis condition

- Agilent Technologies 1200s
- Column: Agilent Eclipse Plus C18 3.5 μm 100 x 4.6 mm
- Flow: 0.3 mL/min
- Detector: PDA detector (210 nm)
- Mobile phase: Water-MeOH gradient

Time	MeOH
0.0	10
30	100
40	100
50	10

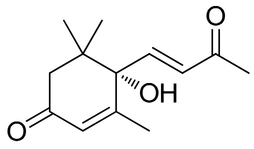

5

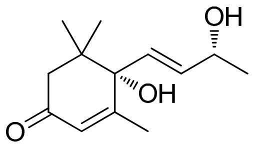

6

Figure S4. LC/MS analysis for purity of compounds **1−6**.

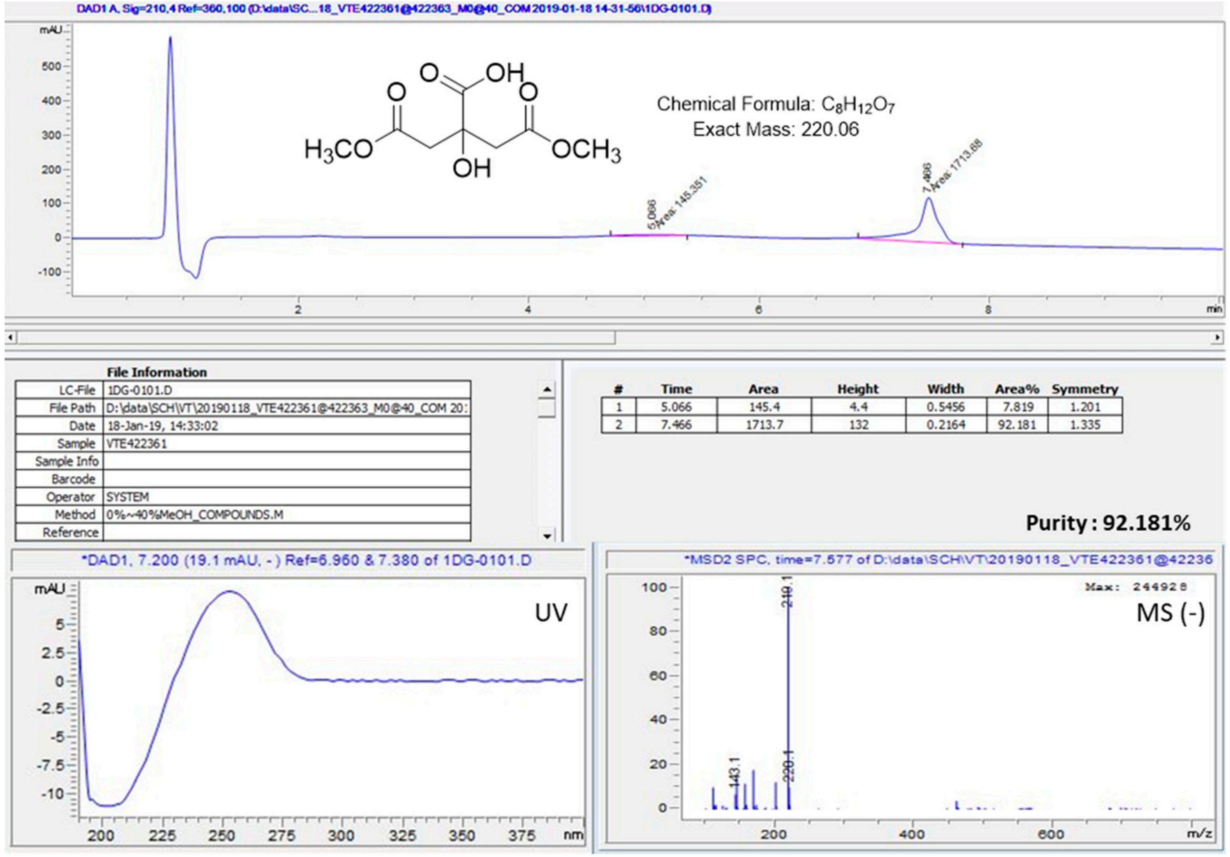

Analysis condition

- Agilent Technologies 1200s
- Column: Phenomenex Kinetex 5 µm C18 100A 100 x 2.1 mm
- Flow: 0.3 mL/min
- Detector: PDA detector (210 nm)
- Mobile phase: Water-MeOH gradient

Time	MeOH
0.0	0
10	40

Compound 1

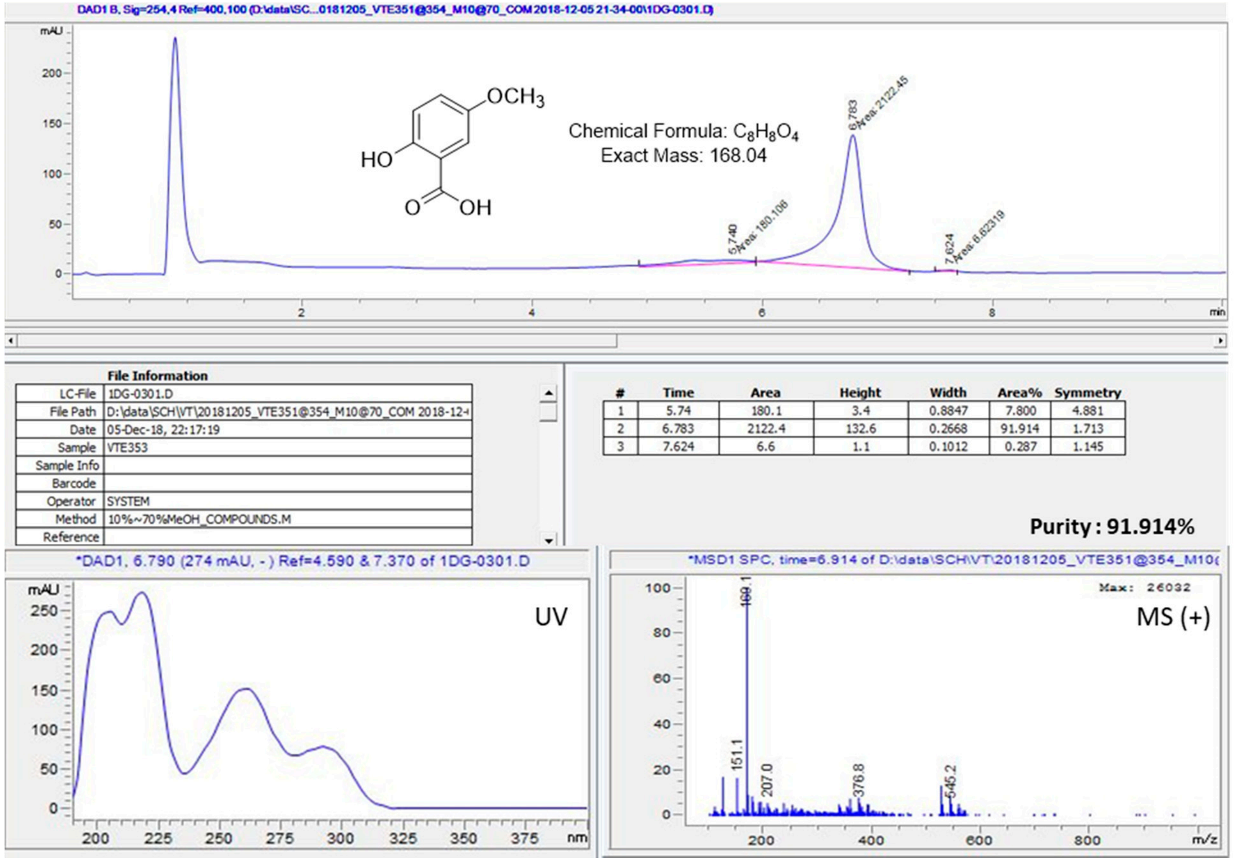

Analysis condition

- Agilent Technologies 1200s
- Column: Phenomenex Kinetex 5 µm C18 100A 100 x 2.1 mm
- Flow: 0.3 mL/min
- Detector: PDA detector (254 nm)
- Mobile phase: Water-MeOH gradient

Time	MeOH
0.0	10
10	70

Compound 2

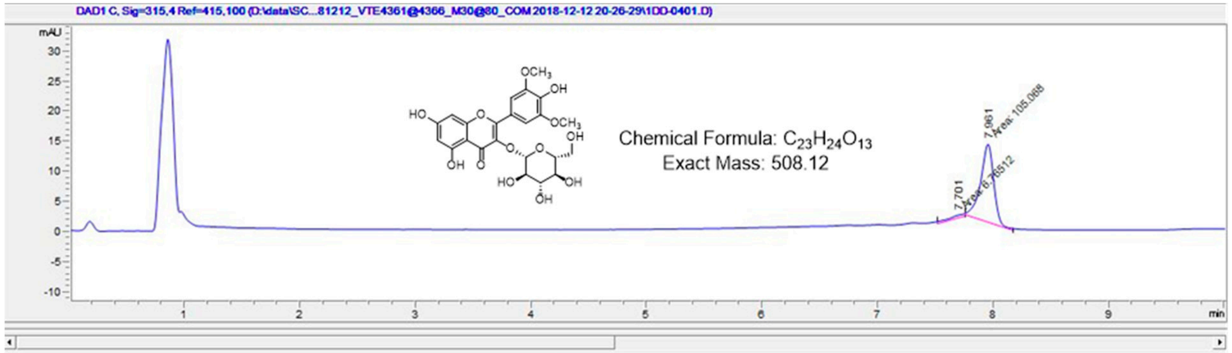

File Information

LC-File	1DD-0401.D
File Path	D:\data\SCH\VT\20181212_VTE4361@4366_M30@80_COM 2018-1
Date	12-Dec-18, 21:30:49
Sample	VTE4364
Sample Info	
Barcode	
Operator	SYSTEM
Method	30%~80%_COMPOUNDS.M
Reference	

#	Time	Area	Height	Width	Area%	Symmetry
1	7.701	6.8	5.8E-1	0.1936	6.049	0
2	7.961	105.1	13.1	0.1342	93.951	1.231

Purity : 93.951%

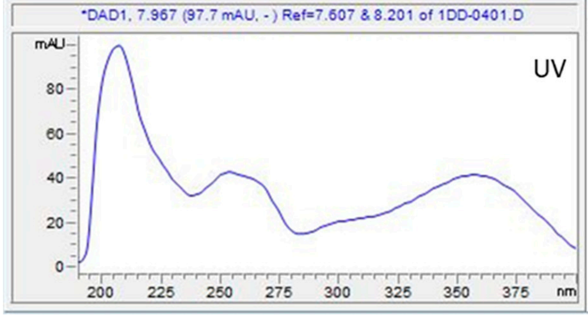

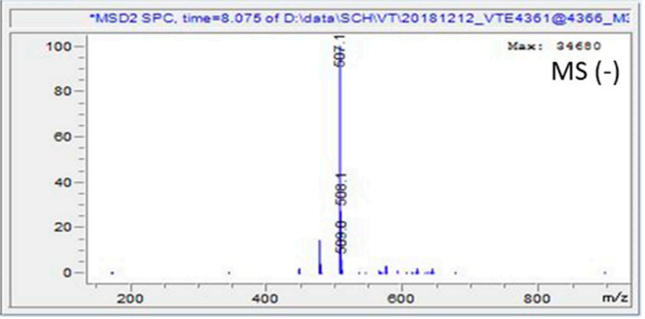

Analysis condition

- Agilent Technologies 1200s
- Column: Phenomenex Kinetex 5 µm C18 100A 100 x 2.1 mm
- Flow: 0.3 mL/min
- Detector: PDA detector (315 nm)
- Mobile phase: Water-MeOH gradient

Time	MeOH
0.0	30
10	80

Compound 3

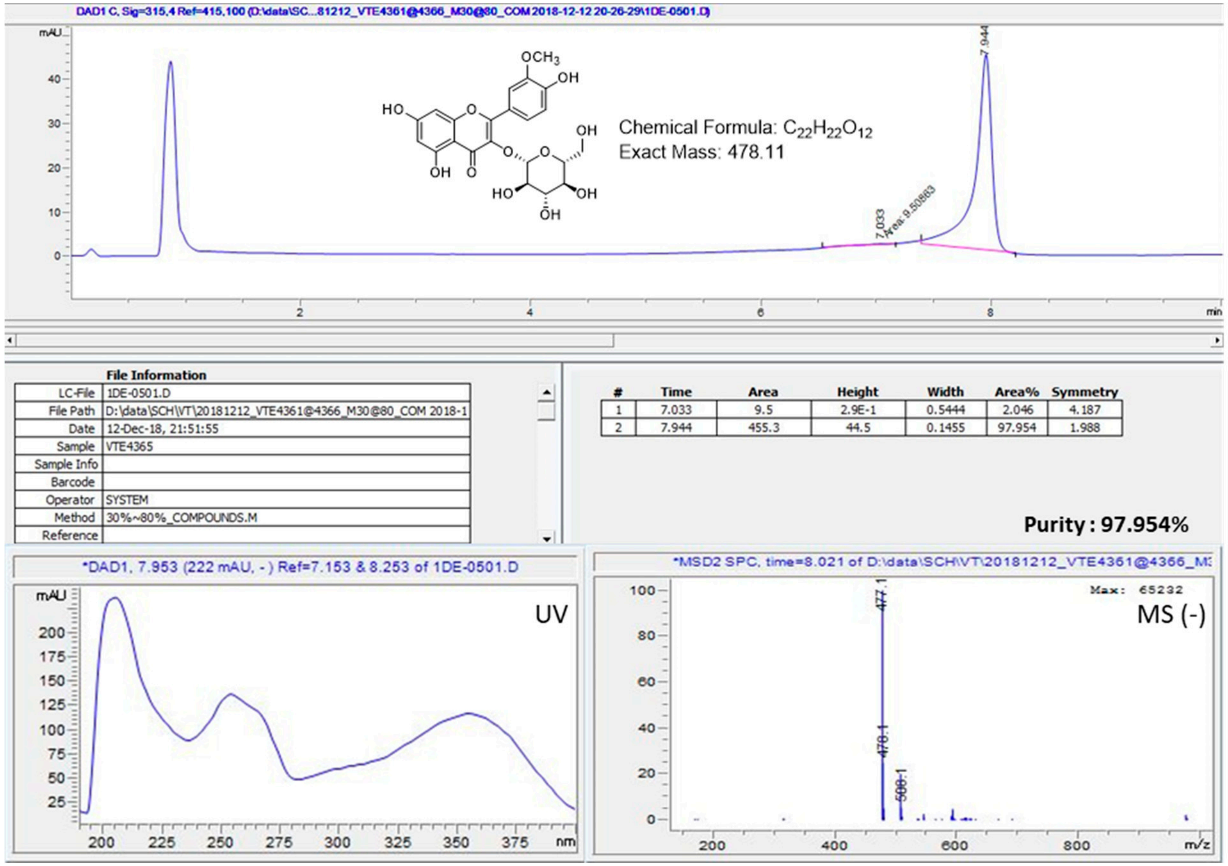

Analysis condition

- Agilent Technologies 1200s
- Column: Phenomenex Kinetex 5 µm C18 100A 100 x 2.1 mm
- Flow: 0.3 mL/min
- Detector: PDA detector (315 nm)
- Mobile phase: Water-MeOH gradient

Time	MeOH
0.0	30
10	80

Compound 4

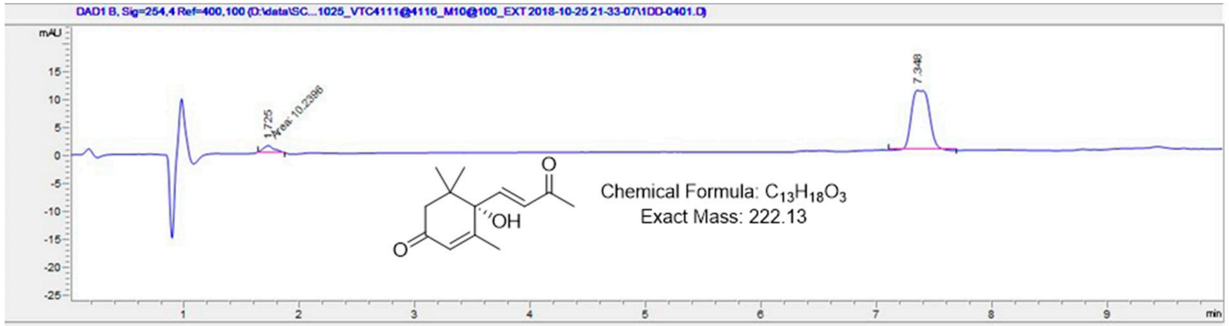

File Information

LC-File	1DD-0401.D
File Path	D:\data\SCH\VT\20181025_VTC4111@4116_M10@100_EXT 2018-:
Date	25-Oct-18, 22:37:35
Sample	VTC4114
Sample Info	
Barcode	
Operator	SYSTEM
Method	10%~100%MeOH_COMPOUNDS.M
Reference	

#	Time	Area	Height	Width	Area%	Symmetry
1	1.725	10.2	1.4	0.1217	8.032	0.869
2	7.348	117.2	10.7	0.1473	91.968	0.518

Purity : 91.968%

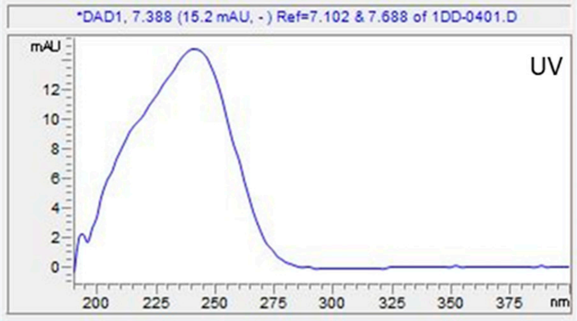

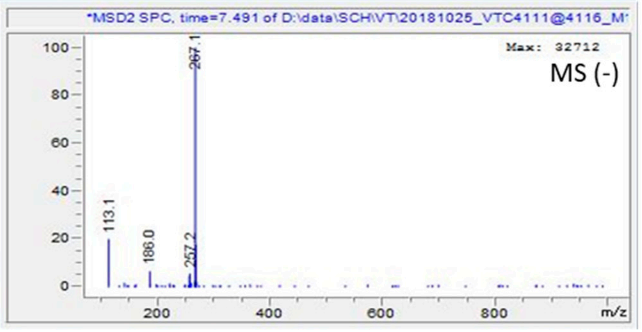

Analysis condition

- Agilent Technologies 1200s
- Column: Phenomenex Kinetex 5 μm C18 100A 100 x 2.1 mm
- Flow: 0.3 mL/min
- Detector: PDA detector (254 nm)
- Mobile phase: Water-MeOH gradient

Time	MeOH
0.0	10
10	100

Compound 5

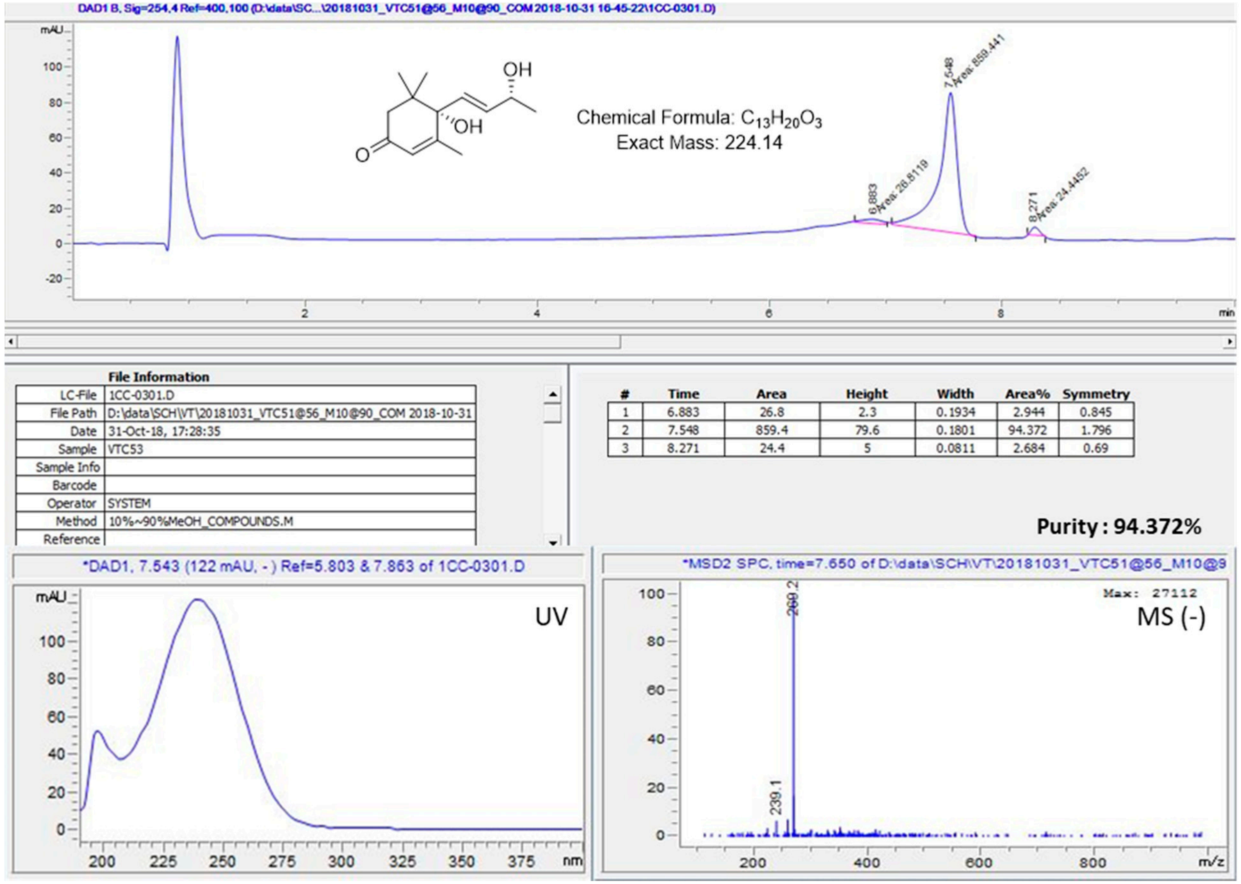

File Information

LC-File	1CC-0301.D
File Path	D:\data\SCH\VT\20181031_VTC51@56_M10@90_COM 2018-10-31
Date	31-Oct-18, 17:28:35
Sample	VTC53
Sample Info	
Barcode	
Operator	SYSTEM
Method	10%~90%MeOH_COMPOUNDS.M
Reference	

#	Time	Area	Height	Width	Area%	Symmetry
1	6.883	26.8	2.3	0.1934	2.944	0.845
2	7.548	859.4	79.6	0.1801	94.372	1.796
3	8.271	24.4	5	0.0811	2.684	0.69

Purity : 94.372%

Analysis condition
- Agilent Technologies 1200s
- Column: Phenomenex Kinetex 5 µm C18 100A 100 x 2.1 mm
- Flow: 0.3 mL/min
- Detector: PDA detector (254 nm)
- Mobile phase: Water-MeOH gradient

Time	MeOH
0.0	10
10	90

Compound 6
